# Phenylbutyrate Counteracts *Shigella* Mediated Downregulation of Cathelicidin in Rabbit Lung and Intestinal Epithelia: A Potential Therapeutic Strategy

**DOI:** 10.1371/journal.pone.0020637

**Published:** 2011-06-03

**Authors:** Protim Sarker, Sultan Ahmed, Snigdha Tiash, Rokeya Sultana Rekha, Roger Stromberg, Jan Andersson, Peter Bergman, Gudmundur H. Gudmundsson, Birgitta Agerberth, Rubhana Raqib

**Affiliations:** 1 International Centre for Diarrheal Disease Research, Bangladesh, Dhaka, Bangladesh; 2 Department of Medical Biochemistry and Biophysics, Karolinska Institutet, Stockholm, Sweden; 3 Department of Biosciences and Nutrition, Center for Infectious Medicine, Karolinska University Hospital Huddinge, Karolinska Institutet, Stockholm, Sweden; 4 Department of Medicine, Center for Infectious Medicine, Karolinska University Hospital Huddinge, Karolinska Institutet, Stockholm, Sweden; 5 Division of Clinical Microbiology, Department of Laboratory Medicine, Karolinska University Hospital Huddinge, Karolinska Institutet, Stockholm, Sweden; 6 Institute of Biology, University of Iceland, Reykjavik, Iceland; National Jewish Health, United States of America

## Abstract

**Background:**

Cathelicidins and defensins are endogenous antimicrobial peptides (AMPs) that are downregulated in the mucosal epithelia of the large intestine in shigellosis. Oral treatment of *Shigella* infected rabbits with sodium butyrate (NaB) reduces clinical severity and counteracts the downregulation of cathelicidin (CAP-18) in the large intestinal epithelia.

**Aims:**

To develop novel regimen for treating infectious diseases by inducing innate immunity, we selected sodium 4-phenylbutyrate (PB), a registered drug for a metabolic disorder as a potential therapeutic candidate in a rabbit model of shigellosis. Since acute respiratory infections often cause secondary complications during shigellosis, the systemic effect of PB and NaB on CAP-18 expression in respiratory epithelia was also evaluated.

**Methods:**

The readouts were clinical outcomes, CAP-18 expression in mucosa of colon, rectum, lung and trachea (immunohistochemistry and real-time PCR) and release of the CAP-18 peptide/protein in stool (Western blot).

**Principal findings:**

Significant downregulation of CAP-18 expression in the epithelia of rectum and colon, the site of *Shigella* infection was confirmed. Interestingly, reduced expression of CAP-18 was also noticed in the epithelia of lung and trachea, indicating a systemic effect of the infection. This suggests a causative link to acute respiratory infections during shigellosis. Oral treatment with PB resulted in reduced clinical illness and upregulation of CAP-18 in the epithelium of rectum. Both PB and NaB counteracted the downregulation of CAP-18 in lung epithelium. The drug effect is suggested to be systemic as intravenous administration of NaB could also upregulate CAP-18 in the epithelia of lung, rectum and colon.

**Conclusion:**

Our results suggest that PB has treatment potential in human shigellosis. Enhancement of CAP-18 in the mucosal epithelia of the respiratory tract by PB or NaB is a novel discovery. This could mediate protection from secondary respiratory infections that frequently are the lethal causes in dysentery.

## Introduction

Antimicrobial peptides (AMPs) are gene encoded antibiotics and important components of the innate defense system, which protect host-microbe interfaces from pathogenic insults [1]. We have earlier shown that the human cathelicidin LL-37 and β-defensin-1 are downregulated in rectal epithelium of patients with shigellosis, suggesting a mechanism facilitating bacterial invasion [2]. This finding was recently confirmed by Sperandio B et al. [3]. Additional pathogens such as *Vibrio cholerae*, enterotoxigenic *Escherichia coli* and *Neisseria gonorrhoeae* also downregulate LL-37 expression in epithelial cells at the respective infection site, indicating a common immune escape strategy by pathogens [2], [4], [5]. Furthermore, LL-37 regulates tissue homeostasis and sustains the integrity of the mucosal barrier as shown in respiratory and intestinal tracts [6], [7].

AMPs are attractive candidates for developing alternative strategies in combating pathogens that become resistant to classical antibiotics. These defense peptides exhibit broad spectrum of antimicrobial activities and low incidence of bacterial resistance [8]. We and others have shown that short chain fatty acids, in particular butyrate, up-regulate LL-37 expression in colonic epithelial cells [9], [10]. In fact, butyrate is a fermentation product of fibers in the colon and hence is present in the lower part of the gut. We further demonstrated that oral treatment with sodium butyrate (NaB) could restore the downregulation of rabbit cathelicidin CAP-18 in the colonic epithelium of *Shigella* infected rabbits with concomitant reduction in clinical severity and bacterial load in stool [11]. However, butyrate is a foul-smelling substance and is thus not suited for oral therapeutic interventions. Recently we discovered that sodium 4-phenyl-butyrate (PB), an odorless derivative of butyrate, is an even more potent inducer of cathelicidin *in vitro* than butyrate [12]. Here we have investigated the *in vivo* efficiency of PB compared to that of NaB treatment by utilizing a rabbit model of shigellosis. Since shigellosis is often associated with secondary respiratory infections [13], [14], [15], we further investigated cathelicidin expression in lung and trachea during *Shigella* infection as well as the remote systemic effect of oral treatment with PB and NaB.

## Results

### Clinical responses of dysenteric rabbits are improved by oral treatment with PB or NaB

**Figure 1:**
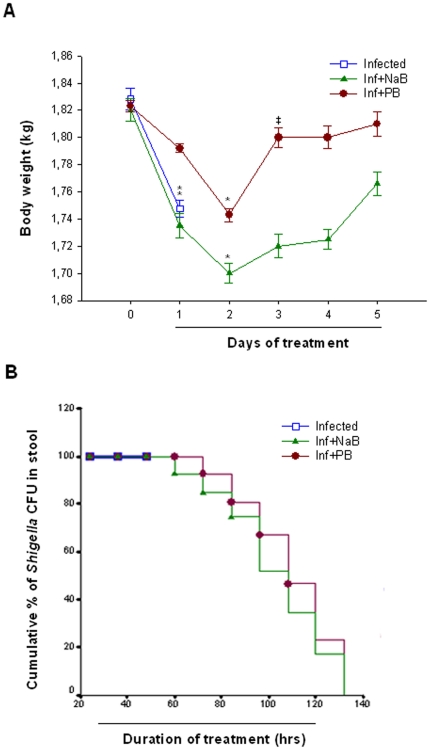
Changes of infection associated events in *Shigella* infected rabbits before, during and after PB/NaB treatment. (A) Body weight loss due to *Shigella* infection was recovered by treatment with PB or NaB. Data are given as mean ± standard deviation of 5 rabbits in each group. Two-way ANOVA with repeated measures on one factor (time) was used in comparing the effects over time between different groups. The differences are significant when P<0.05; *significant loss of body weight compared to day 0 (healthy condition), ^‡^significant recovery compared to day 2. (B) Kaplan-Meier survival plot revealed reduction of *Shigella* CFU in stool of infected rabbits treated with PB (n = 5) or NaB (n = 5).Infected untreated rabbits were sacrificed at day 2. PB: Sodium 4-phenylbutyrate, NaB: Sodium butyrate, CFU: Colony forming unit.

Infected rabbits developed dysentery with thick liquid stool and mucus, occasional blood in stool, reduced body weight, transient fever, lethargy and anorexia, usually after 24 hours of infection. Infected rabbits died within 48 hours if kept untreated. In contrast, treated rabbits survived and recovered from the disease within 3–5 days of treatment as apparent by formed stool and revival from lethargy and anorexia. The initial body weight loss, what was prominent up to 2 days after infection was recovered by treatment with PB or NaB (Fig. 1 A). The recovery of body weight by NaB treatment was steady over time, while PB treatment led to a significant recovery at day 3. Reduction of fever, pus cells, macrophages and red blood cells were comparable in the 2 treatment groups (not shown). Thus, PB appears to ameliorate the clinical symptoms of shigellosis in the rabbit model as demonstrated earlier for NaB [11]. Since untreated infected rabbits could not survive for longer period, the clinical features could not be compared between treated and untreated rabbits.

### *Shigella* load in stool is reduced by oral treatment with PB or NaB

To investigate if the positive effects of PB or NaB treatment on clinical symptoms were connected to reduction of the *Shigella* load in gut, Kaplan-Meier survival plot analysis of colony forming unit (CFU) in stool was performed. However, with PB treatment, most rabbits (4 out of 5) stopped defecating after 2 days of treatment and stool reappeared on 5^th^ day. Therefore, we also included CFU count of rectal swab in this analysis. Treatment with PB or NaB resulted in gradual reduction of *Shigella* shedding over time (Fig. 1 B) and no CFU was observed at day 4 and 5. The effect was parallel in the two treatment group (p = 0.62).

### Release of CAP-18 peptide/protein in stool

**Figure 2:**
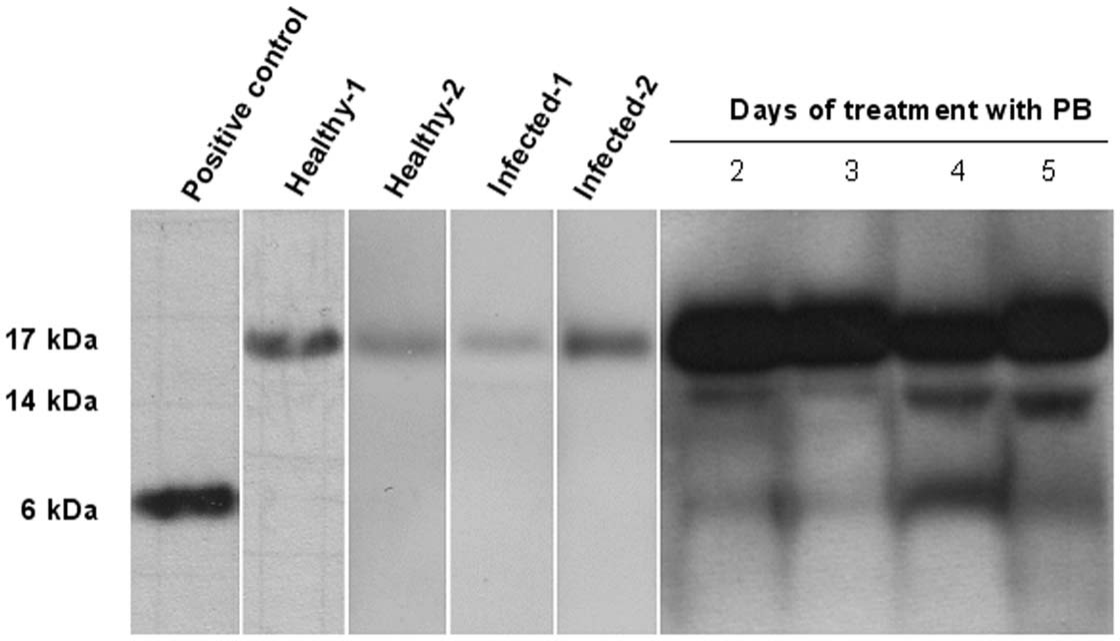
CAP-18 in stool of healthy rabbits, *Shigella* infected rabbits and infected rabbit treated with PB. Western blot analysis of CAP-18 peptide/protein in stool extracts from 2 healthy, 2 *Shigella* infected and 1 infected rabbit treated with PB are shown. The 17 kDa pro-form of CAP-18 was detected in the stool of healthy and infected rabbits before treatment. PB treatment resulted in increased levels of the pro-form and appearance of low levels of the active CAP-18 peptide at day 2 (D2) and day 3 (D3); the level of active peptide was higher at day 4 (D4) that faded at day 5 (D5). Synthetic CAP-18 peptide (1 ng) served as positive control. PB: Sodium 4-phenylbutyrate.

By Western blot analysis of CAP-18 in stool extracts, only pro-form of CAP-18 was observed in healthy and infected rabbits, although band intensity varied between individual rabbits (Fig. 2). As mentioned in the previous section, with PB treatment, stool from only one rabbit could be obtained throughout the treatment regime. In stool extracts from that single rabbit, the pro-form was detected at higher levels from day 2 to 5. The processed form of CAP-18 was also detected at low levels from day 2 to 5 (Fig. 2). In the obtained stool samples at day 1, 2 and 5 from other PB treated rabbits, higher levels of CAP-18 pro-form and low levels of active peptide were also observed after treatment (not shown). The effect of NaB has been demonstrated in our previous study [11] and was reproduced here (not shown).

### CAP-18 peptide/protein is downregulated in the mucosal epithelia of large intestine and respiratory tract after *Shigella* infection

**Figure 3:**
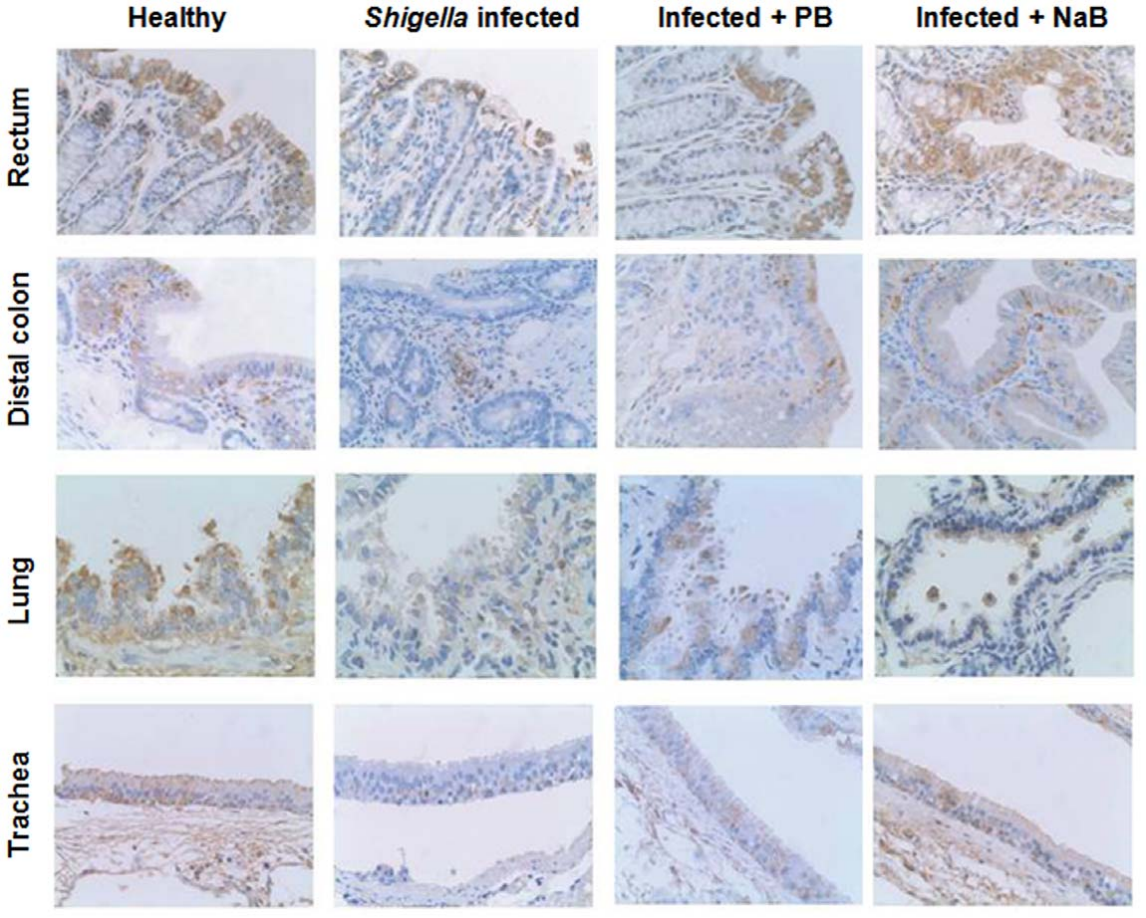
CAP-18 expression in various mucosal tissues of healthy, infected, infected and PB/NaB treated rabbits. Immunohistochemical technique was utilized to detect CAP-18 peptide/protein expression (brown staining) in the mucosal tissue sections of rectum, distal colon, lung and trachea from healthy rabbits, *Shigella*-infected rabbits and infected rabbits treated orally with PB or NaB. Representative pictures are shown. Original magnification was ×400 for all except for lung which was ×600. **Row 1.** The expression of CAP-18 in enterocytes in the surface epithelium of rectum was reduced in infected rabbit compared to healthy rabbit. CAP-18 expression in the surface epithelium of rectum in infected rabbit increased after treatment with PB or NaB. **Row 2.** CAP-18 expression in the distal colon was decreased in enterocytes in the surface epithelium in infected rabbit compared to healthy rabbit. NaB amplified the CAP-18 expression in the surface epithelium of colon in infected rabbit, while PB exhibited no apparent effect. **Row 3.** In lung, CAP-18 expression was located in the ciliated and mucus cells in the epithelial layer and in the alveolar macrophages. Decreased expression of CAP-18 was observed in the lung epithelial layer of infected rabbit compared to healthy rabbit. PB or NaB treatment led to increased expression of CAP-18 peptide in the lung epithelial lining of infected rabbit. **Row 4.** CAP-18 expression was localized to the ciliated epithelium of the trachea. CAP-18 peptide was downregulated in the tracheal epithelium of infected rabbit in comparison with healthy rabbit. Treatment with NaB counteracted this downregulation, while treatment with PB had no obvious effect. PB: Sodium 4-phenylbutyrate, NaB: Sodium butyrate.

**Figure 4:**
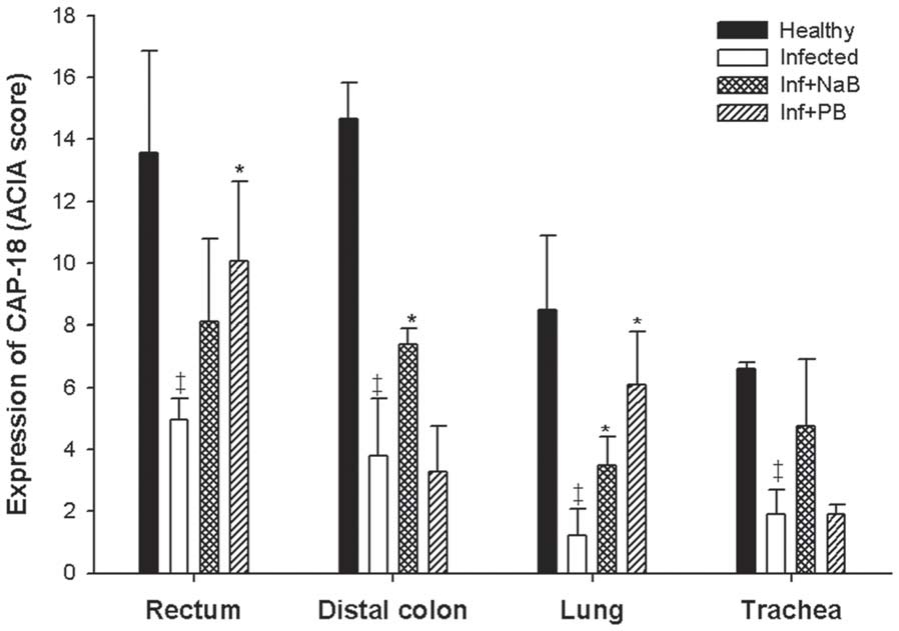
CAP-18 immunoreactivity in various mucosal epithelia of healthy, infected, infected and treated with PB/NaB rabbits. Mucosal sections of rectum, distal colon, lung and trachea from healthy rabbits (n = 5), *Shigella*-infected rabbits (n = 5) and infected rabbits treated orally with NaB (n = 5) or PB (n = 5) were stained with CAP-18 specific antibody. Quantification of immunoreactive area relative to the total cell area of the epithelia was done by a computerized image-analysis technique, and the results are expressed as ACIA score, i.e., the total positively stained area×total mean intensity (1–256 levels/per pixel) of positive area divided by total cell area (See materials and methods). Data are given as mean ± standard deviation. One-way ANOVA (or ANOVA on ranks for data that were not normally distributed as assessed by Q-Q plot and histogram) was used in comparing different groups of rabbits. The differences are significant when P<0.05; ^‡^significant when compared to healthy, *significant when compared to infected. PB: Sodium 4-phenylbutyrate, NaB: Sodium butyrate.

Immunohistochemical analyses (Fig. 3 and 4) revealed significant downregulation of CAP-18 peptide/protein after *Shigella* infection in the epithelia of rectum (p = 0.008) and distal colon (p≤0.001) compared to healthy rabbits. This is in line with our previous result [11]. Interestingly, we also detected a significant downregulation of epithelial CAP-18 in the lung (p = 0.001) and trachea (p = 0.016) after *Shigella* infection compared to healthy rabbits. No obvious changes in CAP-18 peptide/protein expression were observed in the non-epithelial region of the organs investigated (Fig. 3).

### CAP-18 peptide/protein is enhanced in the epithelia of large intestine and respiratory tract in *Shigella* infected rabbits after oral treatment with PB or NaB

In the rectal epithelium, treatment with PB significantly (p = 0.004) counteracted the *Shigella* mediated downregulation of CAP-18 peptide/protein, while the effect of NaB was prominent although not significant (p = 0.07) compared to infected untreated rabbits (Fig. 3 and 4). In the epithelium of distal colon, NaB treatment significantly upregulated CAP-18 peptide/protein expression as compared to infected untreated rabbits (p = 0.003), while PB exhibited no apparent effect.

Notably, oral PB (p = 0.002) and NaB (p = 0.024) treatment significantly enhanced CAP-18 peptide/protein in the lung epithelium compared to infected untreated rabbits (Fig. 3 and 4). Moreover, treatment with NaB resulted in higher expression of CAP-18 peptide/protein in the tracheal epithelium compared to infected untreated rabbits, though the difference was not significant. No change was evident in the tracheal epithelium after PB treatment.

Thus, PB exhibits higher potency than NaB in inducing CAP-18 peptide/protein expression in the epithelia of rectum and lung, while the effects on colonic and tracheal epithelia are not obvious.

### NaB systemically induces CAP-18 peptide/protein expression in the epithelia of large intestine and respiratory tract

**Figure 5:**
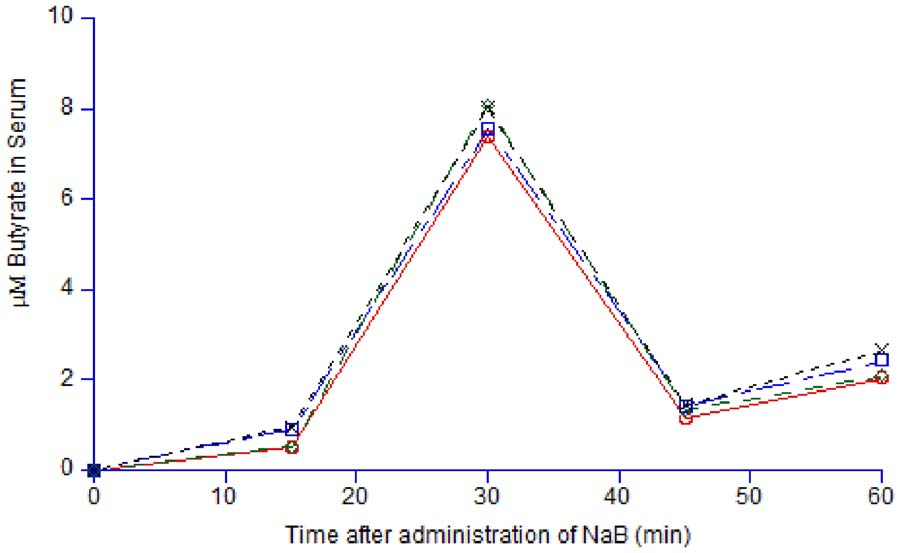
Butyrate concentration in healthy rabbit serum over time after oral treatment with NaB. Serum was collected from healthy rabbits at different time points after oral treatment with a single 0.14 mmol dose of NaB. Four analyses (duplicate analyses of two samples) of serum from a NaB treated rabbit are shown, where the concentrations are calculated from two separate standard curves (one for each duplicate). NaB: Sodium butyrate.

**Figure 6:**
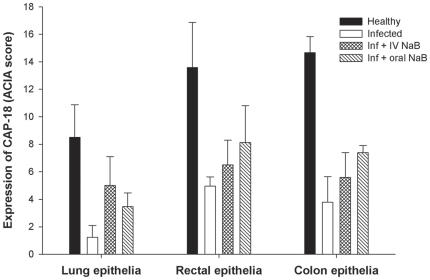
Comparison between intravenous and oral NaB treatment of *Shigella* infected rabbits on epithelial CAP-18 immunoreactivity. Immunostaining of CAP-18 peptide/protein was done in the tissue sections of rectum, distal colon and lung from healthy rabbits (n = 5), *Shigella*-infected rabbits (n = 5), infected rabbits treated with NaB intravenously (IV) (n = 2) or orally (n = 5). A computerized image-analysis technique was applied to quantify the immunoreactive area relative to the total cell area of the epithelia and the results are expressed as ACIA score, i.e., the total positively stained area×total mean intensity (1–256 levels/per pixel) of positive area divided by total cell area (See materials and methods). Data are given as mean ± standard deviation. NaB: Sodium butyrate.

Butyrate is known to disseminate into blood after oral treatment with NaB [16]. We also detected butyrate in rabbit serum in particular at 30 minutes after oral treatment with a single 0.14 mmol dose of NaB (Fig. 5). Moreover, intravenous injection of NaB into *Shigella* infected rabbits induced CAP-18 peptide/protein expression in the epithelia of lung, rectum and colon (Fig. 6).

### The levels of CAP-18 transcripts during *Shigella* infection and after oral treatment with PB or NaB

In an attempt to support our immunhistochemical data based on ACIA-scores, mRNA levels in intestinal and respiratory tissue extracts were measured. However, in contrast to the effect on the peptide/protein level, *Shigella* infection resulted in increased CAP-18 transcripts in lamina propria (LP) as well as in surface epithelium (SE) of distal colon compared to healthy controls. The increase was significant in LP (p = 0.006) but not in SE (p = 0.172). Treatment with NaB significantly normalized the *Shigella* mediated mRNA accumulation in both LP (p = 0.006) and SE (p = 0.008) in the colon, whereas the effect of PB was significant only in LP (p = 0.016) compared to infected untreated rabbits. No obvious changes in CAP-18 transcript levels were observed after infection as well as after treatment with PB or NaB in both SE and LP of rectum (Table 1).

**Table 1:** CAP-18 gene expression in the tissue specimens from various organs of healthy rabbits, *Shigella*-infected rabbits and infected rabbits treated with PB or NaB.

Specimens	Healthy rabbits	Infected rabbits	Infected rabbits treated with	
	(n = 5)	(n = 5)	PB (n = 5)	NaB (n = 5)
Distal Colon, SE	11.6 (8.1–16.1)	22.2 (14.8–25.2)	14.3 (2–29)	5.02 (2.7–5.6)^b^
Distal Colon, LP	0.42 (0.31–0.73)	19.7 (14.3–31.8)^a^	0.53 (0.23–1.04)^b^	0.55 (0.47–0.83)^b^
Rectum, SE	0.77 (0.44–1.11)	0.93 (0.52–1.35)	0.18 (0.17–0.19)	0.3 (0.18–0.42)
Rectum, LP	0.18 (0.14–0.41)	0.12 (0.09–0.16)	0.41 (0.27–0.59)	0.1 (0.08–0.11)
Lungs	0.8 (0.65–2.11)	581.2 (530.9–735.5)^a^	5.3 (2.7–8)^b^	12.6 (10.4–15)^b^
Trachea	1.14 (0.52–1.81)	36.3 (18.4–61.9)^a^	1.1 (0.43–1.3)^b^	0.71 (0.32–1.82)^b^

Data are expressed as median with 25 and 75 percentiles in parentheses. Healthy control rabbits are without any treatment. One-way ANOVA on ranks was used in comparing data between different groups of rabbits. The differences are significant when P<0.05;

a significant when compared to healthy,

b significant when compared to infected. SE: Surface epithelium, LP: Lamina propria, PB: Sodium 4-phenylbutyrate, NaB: Sodium butyrate.

During shigellosis, CAP-18 mRNA was increased significantly in lung (p = 0.006) and trachea (p = 0.001) compared to healthy controls. The levels of mRNA in both lung and trachea was significantly normalized after treatment with PB (p = 0.029 for lung, p = 0.004 for trachea) or NaB (p = 0.029 for lung, p = 0.001 for trachea) (Table 1).

### Oral treatment with PB confers no toxic effect in rabbits

Since PB is a potential drug candidate for treatment of infectious diseases, renal and hepatic biomarkers for toxicity were monitored. No significant differences were observed in the plasma levels of these biomarkers between healthy, healthy rabbits treated with PB, infected and infected rabbits treated with PB (Table 2). Similar results were observed earlier after NaB treatment of *Shigella* infected rabbits [11].

**Table 2:** Levels of hepatic and renal markers in plasma of healthy, *Shigella* infected and healthy/infected rabbits treated with phenylbutyrate.

Rabbits	Markers for kidney function		Liver enzymes	
	Creatinine (µmol/dL)	Urea (mg/dL)	ALT (U/L)	γ-GT (U/L)
Healthy	52.3±27.6	32.5±10.8	46.7±15.9	7.5±4.0
Healthy+PB	67.8±14.5	25.3±4.9	35±4.9	7.6±2.7
Infected	209.1±203.1	113.8±70.8	75.3±75.3	8.9±2.2
Infected+PB	68.4±5.0	31.6±19.5	39.5±4.1	7.1±3.8

Data are given as mean ± standard deviation. ALT Alanine transaminase; γ-GT, gamma glutamyl transferase. Plasma samples were collected after 3 days of twice daily treatment with phenylbutyrate. Normal reference value of creatinine, urea, ALT and γ-GT for rabbits are 53–70.7 µmol/dL, 30–37.3 mg/dL, <41 U/L and 0–14 U/L, respectively.

## Discussion

In the current study, we demonstrate for the first time that acute *Shigella* infection despite being mostly a large intestinal infection also downregulates the cathelicidin CAP-18 in the lung and tracheal epithelial surfaces in a rabbit model. Treatment with phenylbutyrate (PB) resulted in clinical recovery from shigellosis, with clearance of *Shigella* from stool after 3–4 days of treatment. Oral PB treatment counteracted the downregulation of CAP-18 peptide/protein in the rectal as well as in lung epithelia. PB is approved as a drug for urea cycle disorder. Therefore, we propose that PB can be evaluated in pharmaceutical intervention of human shigellosis.

Our group first demonstrated that *Shigella* downregulates human cathelicidin expression in epithelia of rectal biopsies and in colonic epithelial and monocytic cell lines [2]. Interestingly, purified plasmid DNA was indicated as the mediator of the downregulation. Later, Sperandio et al. suggested plasmid-encoded effector proteins, regulated by MxiE transcriptional activator being responsible for *Shigella* mediated downregulation of innate effectors including LL-37 [3]. However, the exact cell signaling pathways, mediating downregulation of LL-37 have not yet been resolved, although MxiE-driven effectors have been shown to target NF-κB and MAPK signaling pathways [17], [18], [19].

We have previously shown that oral intake of sodium butyrate (NaB) by *Shigella* infected rabbits led to reduced clinical severity, reduced bacterial load in stool along with restitution of CAP-18 peptide/protein in the large intestinal epithelia and the conversion of the pro-form of CAP-18 into its active form in stool [11]. Here, we show that PB, a derivative of butyrate also mediated clinical recovery from shigellosis. Oral PB treatment could also reduce *Shigella* shedding in stool. By Western blot analysis, the active form of CAP-18 was detected in stool after treatment with PB, which supports the involvement of CAP-18 in *in vivo* bactericidal activity, although additional antimicrobial components may also be involved. However, unlike NaB, which upregulated CAP-18 peptide/protein expression in both rectal and colonic epithelia, PB upregulated CAP-18 only in rectal epithelium, indicating a site-specific activity of PB. Given the increase of CAP-18 proform in Western blot analysis of stool from PB treated rabbits, it is worth noting that PB treatment not only recovers tissue levels of CAP-18 expression in the intestine but also significantly effects the secretion of this antimicrobial peptide.

Earlier epidemiological studies have reported that dysentery and cholera-like diarrhea outbreaks are often associated with secondary complications such as pneumonia and meningitis with a fatal outcome [20], [21]. In hospital based studies of patients with dysentery or diarrhea, bronchopneumonia has often been the underlying associated cause of death [13], [14], [15], [22]. Here we report a novel discovery that *Shigella* infection causes downregulation of the antimicrobial peptide CAP-18 in lung and tracheal epithelia. The *Shigella* associated downregulation of CAP-18 suggests a functional decline in the innate epithelial barrier of the respiratory system, facilitating invasion by respiratory pathogens. This may partially explain the frequent association of pneumonia with shigellosis. The *in vitro* killing of respiratory pathogens, *Haemophilus influenzae* and *Moraxella catarrhalis* by CAP-18 (unpublished data) further supports this notion.

In the present study, we show that downregulation of CAP-18 peptide/protein was counteracted in lung epithelium, upon oral intake of PB or NaB. Thus, treatment with these substances seems to maintain expression of critically active components of the innate defense barrier. Detection of butyrate in rabbit serum after oral treatment with NaB suggest that orally administered NaB or PB are absorbed from the intestine and reach the mucosa of various organs via the blood stream and influence CAP-18 expression in the remote organs. Indeed, we found that intravenous injection of *Shigella* infected rabbits with NaB also counteracted the downregulation of CAP-18 peptide/protein in the epithelia of rectum, colon and lung.

Cellular pathways of cathelicidin induction by butyrate have been studied in several human cell lines or primary cultures. The common denominator for the induction by butyrate is its activity as histone deacetylase inhibitor (HDACi), facilitating transcription [23], [24]. The involvement of MAPK signaling pathways, nuclear hormone receptors and transcription factor binding sites have also been demonstrated [10], [23], [25], [26]. Our group has recently shown that, phenylbutyarte also involves activation of MAPK in lung epithelial cells for *CAMP* gene (LL-37 encoding gene) induction [12]. However, this study showed that the HDACi activity of PB is not due to a direct effect on the chromatin structure at the *CAMP* proximal promoter. Instead, enhanced histone acetylation facilitates expression of other genes, encoding critical factors, regulating *CAMP* gene expression. An alternative pathway has also been shown for the effect of butyrate, involving G-protein coupled receptor (GPCR). Binding of short chain fatty acids including butyrate to this receptor has been shown to affect inflammatory and immune responses [27], [28]. Butyrate-GPCR interaction might also be involved in the induction of cathelicidin, an event that needs to be addressed.

The levels of CAP-18 transcripts were enhanced in distal colon, lung and trachea in *Shigella* infected rabbits compared to healthy controls. These findings did not correlate with the CAP-18 peptide/protein expression in the mucosal epithelia of these organs. We have earlier observed a similar finding in the rectum of patients with shigellosis, where transcripts of several cytokines were 3–100 fold higher compared to the corresponding protein expression [29]. These findings suggest a bacterial or host mediated post-transcriptional regulation of certain cytokines and in the present case also CAP-18. Shiga toxin or shiga-like cytotoxins, which are known to inhibit host cell protein synthesis [30], [31] might be responsible for the observed low expression of CAP-18 peptide/protein and accumulation of mRNA. Translational arrest at initiation and elongation has also been demonstrated in influenza virus and adenovirus infections [32]. In our earlier study of patients with shigellosis, the downregulation of LL-37 transcripts in the gut mucosa was observed in the acute stage and the proportion of patients with this downregulation increased in the early convalescent stage [2]. In fact, the acute stage biopsies were taken from patients between 3–5 days after onset of diarrhea and thus it was not possible to evaluate whether there was an initial upregulation followed by a decline in LL-37 transcripts in the patients. In rabbits, the biopsies were collected within 24 hour of infection; we could not keep the infected rabbits alive for longer period to monitor the status of CAP-18 transcripts without treatment. The production of high levels of CAP-18 mRNA reflects a strategy of the host to intensify the innate defense to protect against the invading pathogens. However, pathogens may in someway interfere with the translation and thereby escape host defense. Notably, after treatment with PB or NaB, the increase of transcripts during infection was normalized, while the peptide/protein expression was increased. The detailed mechanisms of this post-transcriptional regulation need to be elucidated.

In summary, our results suggest phenylbutyrate to be a drug candidate against shigellosis through its ability to counteract the *Shigella* mediated downregulation of the gut's innate epithelial barrier. This oral treatment may also reinforce remote mucosal immunity of the respiratory tract to combat secondary respiratory infections, which are frequently associated with dysentery or diarrhea. Further studies are underway to evaluate the potential uses of phenylbutyrate in respiratory diseases.

## Materials and Methods

### Ethics statement

The study (Research protocol # 2007-065) was approved by the Animal Experimentation Ethics Committee (AEEC) of the International Centre for Diarrheal Disease Research, Bangladesh (ICDDR,B) [May 07, 2008]. Based on the recommendations in the Guide for the Care and Use of Laboratory Animals of the National Institutes of Health (NIH), ICDDR,B developed its own rules and guidelines. These guidelines and the subsequent modifications were approved by the ICDDR,B Board of Trustees.

### Bacterial strain

An invasive clinical isolate of *Shigella flexneri* 2a as tested by the Serény test and Congo red binding assay was used for infecting rabbits [11].

### Candidate compounds

Sodium butyrate (NaB) and sodium 4-phenylbutyrate (PB) were obtained from Sigma-Aldrich, Steinheim, Germany.

### Antimicrobial peptide CAP-18 and the corresponding specific antibody

Synthetic CAP-18 peptide (GLRKRLRKFRNKIKEKLKKIGQKIQGLLPKLAPRTDY) (Innovagen, Lund, Sweden) is the rabbit homologue of the human cathelicidin LL-37. Affinity purified polyclonal chicken antiserum against CAP-18 (Innovagen) recognizes both the proform and the mature CAP-18 peptide. The proform/mature CAP-18 is designated as CAP-18 protein/peptide in the manuscript.

### Animal model

Inbred New Zealand white rabbits of either sex weighing 1.8–1.9 kg were maintained in the animal resource facilities of ICDDR, B. Healthy rabbits free of enteric pathogens e.g., *Salmonella*, *Shigella*, *Vibrio cholera* and *Coccidia* were studied. Rabbits were infected with *S. flexneri* 2a to develop shigellosis. The infected rabbits (n = 15) were orally treated with PB (n = 5) or NaB (n = 5) or left untreated (n = 5). Expression of the CAP-18 protein/peptide and mRNA in various specimens was analyzed in all the infected and infected-treated rabbits and compared with healthy untreated rabbits (n = 5). Biosafety evaluation was done in infected rabbits, infected rabbits treated with PB, healthy rabbits treated with PB (n = 5) and compared with healthy untreated rabbits (n = 5).

### Infection and treatment procedure

A nonsurgical rabbit model of shigellosis was used as described [11]. Bacterial suspension [10^9^ cfu in 7 ml of normal saline] was given via a sterile feeding tube to each rabbit. After development of dysenteric symptoms (usually within 24 h of inoculation), rabbits of the treatment groups were given NaB (0.14 mmol/dose/rabbit) or PB (0.14 mmol/dose/rabbit) in 7-ml of normal saline (pH 7.2) orally twice daily for 5 consecutive days. Thereafter, rabbits were sacrificed with an overdose of intravenous sodium pentobarbital (66 mg/kg body weight; Sigma). Infected untreated rabbits were sacrificed when the sufferings seemed intolerable, usually on the 2^nd^ day. Healthy rabbits were treated similarly with PB or left untreated. The initial oral dose of butyrate (0.14 mmol/dose/rabbit) was based on previous study [11]. The same dose was selected for PB.

### Specimen collection

Stool samples were collected before inoculating bacteria, after development of dysenteric symptoms, and twice daily during treatment. In parallel, rectal swabs were plated onto MacConkey agar plates and incubated overnight at 37°C. After the rabbits were sacrificed, blood was collected immediately by puncturing the heart and serum and plasma were stored at −80°C. Tissue biopsies of distal colon, rectum, lung and trachea were stored in 10% buffered formalin for immunohistochemical evaluation. For quantitative real-time PCR, tissue specimens from lung and trachea were collected directly in Trizol (Gibco-BRL, Auckland, New Zealand). For colon and rectum, surface epithelia (SE) and lamina propria (LP) were separated to discriminate between epithelial cells and infiltrating immune cells. The separation was performed using a previously described protocol [33] with slight modifications. Briefly, tissue biopsies were washed extensively with Ca^++^/Mg^++^ free PBS (CMF-PBS, pH 7.2) containing penicillin-streptomycin. Tissues were then cut into small pieces followed by incubation in extraction buffer containing 1 mM EDTA, penicillin-streptomycin in CMF-PBS under stirring for 30 min at 37°C. Detached epithelial cell suspension was spun down and stored in Trizol at −80°C. Lamina propria cells were also stored in Trizol.

### Clinical efficacy and biosafety of treatment

Clinical recovery of the rabbits from shigellosis was established as described [11]. For biosafety evaluation, plasma collected after the final treatment and from untreated rabbits were assessed for the levels of hepatic enzymes (alanine transaminase and γ-glutamyl transferase) and renal markers (urea and creatinine) as performed earlier upon butyrate treatment [11].

### Bacterial count in stool

Bacterial load in stool was quantified by plating serial dilutions of stool onto MacConkey agar plates and colonies were counted after an overnight incubation at 37°C. The results were expressed as colony forming units (CFU) per gram of stool. Rectal swab was plated directly onto plate and colonies were counted after overnight incubation.

### Stool extraction and enrichment of peptide/protein

Stools were diluted 10 times with 60% acetonitrile in 1% aqueous trifluoroacetic acid (TFA) and extracted overnight at 4°C. The extracts were centrifuged and supernatants were passed through a 0.45- µm filter and lyophilized. The lyophilized extracts were dissolved in aqueous 0.1% TFA, enriched for peptides and proteins by utilizing OASIS cartridges (Waters, Milford, Massachusetts) as described [11] and again lyophilized. These peptide/protein concentrates were used for Western blot analyses.

### Western blot

Enriched peptide/protein extracts (approximately 30 µg) from stool samples were separated with sodium dodecylsulfate-polyacrylamide gel electrophoresis (SDS-PAGE), utilizing 4–12% NuPAGE Ready Gels (Invitrogen, Carlsbad, CA, USA) followed by electrophoretic transfer onto polyvinyldifluoride (PVDF) membranes. Immunoreactivity was detected by subsequent incubation of the membrane with affinity purified chicken polyclonal CAP-18 antibody (0.2 µg/ml) and donkey-anti-chicken IgY conjugated with horseradish peroxidase (Jackson Immunoresearch Laboratories Inc., West Grove, PA, USA). The enhanced chemiluminescence (ECL) Western blot detection system (GE Healthcare, Buckinghamshire, UK) was used to visualize the bands.

### Immunohistochemistry

Formalin fixed tissue pieces were embedded in paraffin and cut into 3 micron thick sections. Sections were deparaffinized and stained with the affinity purified chicken polyclonal CAP-18 antibody (6.8 µg/ml) (Innovagen) as described [11]. To control for specific staining, synthetic CAP-18 was incubated at 20-fold-higher concentration with the CAP-18 antibody overnight at 4°C, and the mixture was used as above for immunostaining.

### Image analyses

Immunohistochemical staining of CAP-18 *in situ* was analyzed by using a microscope (Leica Microsystems GmbH, Wetzlar, Germany) and the image analysis system Quantimate Q550 (Leica). The epithelial and non-epithelial areas were separately assessed for quantification of CAP-18 staining in each tissue section at 400× magnification and the results were given as ACIA (Acquired Computerized Image Analysis) score [34] (see Supporting Information S1 for details).

### Real-Time RT-PCR

RNA was extracted from biopsy specimens in Trizol, according to the manufacturer's instructions (Qiagen GmbH, Hilden, Germany). Corresponding cDNA was synthesized using Superscript III First-Strand Synthesis System (Invitrogen). CAP-18 transcripts, relative to the housekeeping 18S rRNA were measured in triplicate from the cDNA samples by real-time quantitative RT-PCR, using an Applied Biosystems PRISM model 7700 sequence detection instrument (Applied Biosystems, Foster City, CA, USA) and the 18S rRNA -housekeeping kit (Applied Biosystems). The levels of 18S rRNA did not differ between controls, infected and treated rabbits and was thus suitable as housekeeping gene. The sequences of forward and reverse primers for CAP-18 transcript were 5′-GGAAGATGGGCTGGTGAAGC-3′and 5′-GCGCAGCCCAGTAGGTTCTG-3′, respectively (Primer Express; Applied Biosystems). The TaqMan fluorogenic probe used for CAP-18 was 6-FAM-CAACAGGGCCCAAGAG-MGB (Applied Biosystems). The results were analyzed by using the relative standard method [5].

### Butyrate in blood

To ensure that butyrate was disseminated via blood to act on remote mucosal sites, *Shigella* infected rabbits (n = 2) were injected intravenously with 0.07 mmol/dose/rabbit of NaB (half of the daily oral dose), twice daily for 3 days. Rectal, colonic and lung tissues were collected and processed for CAP-18 immunostaining.

In addition, healthy rabbits (n = 2) were orally treated with NaB (0.14 mmol/dose/rabbit) and blood was collected from the ear vein 15, 30, 45 and 60 minutes after treatment. Butyrate levels in serum were determined by GC-MS based on the method by Su et al. [35] with minor modifications (Supporting Information S2).

### Statistical analyses

Statistical analyses were performed by using SigmaStat 3.1 for Windows (Systat Software Inc., Point Richmond, CA, USA) and SPSS 12.0 for Windows (SPSS Inc, Chicago, Illinois, USA). Data were expressed as mean with standard deviation or median with 25–75 percentiles. Probabilities were regarded as significant when P<0.05. *Shigella* CFU in stool was stated using Kaplan–Meier plot and were compared between groups by using the log-rank (Mantel–Cox) test. Clinical recovery over time between different groups was analyzed by two-factor (treatment and time) ANOVA with repeated measures on one factor (time). Differences in CAP-18 immunostaining and CAP-18 transcripts among different groups were analyzed by one-way ANOVA. For data that were not normally distributed (as assessed by Q-Q plot and histogram), ANOVA on ranks was applied.

## Supporting Information

Supporting Information S1 **Computerized image analysis for detection of immunostaining.**
(DOC)

Supporting Information S2 **Analysis of butyrate in serum.**
(DOC)
