# Supplementary material for: Phenylbutyrate Counteracts Shigella Mediated Downregulation of Cathelicidin in Rabbit Lung and Intestinal Epithelia: A Potential Therapeutic Strategy
Source: PLoS One. 2011 Jun 3;6(6):e20637. doi: 10.1371/journal.pone.0020637 (PMC3108617; doi:10.1371/journal.pone.0020637)
Supplement: Supporting Information S1 — Computerized image analysis for detection of immunostaining. (DOC) [file pone.0020637.s001.doc]

**Supporting Information S1**

for

**Phenylbutyrate Counteracts *Shigella* Mediated Downregulation of Cathelicidin in Rabbit Lung and Intestinal Epithelia: A Potential Therapeutic Strategy**

Protim Sarker^1,2^, Sultan Ahmed^1^, Snigdha Tiash^1^, Rokeya Sultana Rekha^1,2^, Roger Stromberg^3^, Jan Andersson^4^, Peter Bergman^4,5^, Gudmundur H. Gudmundsson^6^, Birgitta Agerberth^2,a^, Rubhana Raqib^1,a^

^1^International Centre for Diarrheal Disease Research, Bangladesh, 1212 Dhaka, Bangladesh; ^2^Department of Medical Biochemistry and Biophysics, Karolinska Institutet, 17177 Stockholm, Sweden  ^3^Departments of Biosciences and Nutrition, ^4^ Department of Medicine, Center for Infectious Medicine (CIM), ^5^Department of Laboratory Medicine, Division of Clinical Microbiology, Karolinska University Hospital Huddinge, Karolinska Institutet, 14186 Stockholm, Sweden; ^6^Institute of Biology, University of Iceland, 101 Reykjavik, Iceland.

**Computerized image analysis for detection of immunostaining**

Acquired computerized image analysis was used to quantify immunohistochemical staining *in situ* according to Cunnane G et al. (Cunnane G, Bjork L, Ulfgren AK, et al. Quantitative analysis of synovial membrane inflammation: a comparison between automated and conventional microscopic measurements. Ann Rheum Dis 1999;58:493-9). Digital images of the stained tissue samples were transferred from a microscope (Leica Microsystems GmbH, Wetzlar, Germany) to a computerized image analysis system Leica Qwin Runner (version 3) through a three-chip charged-coupled device (CCD) color camera DCF295 (Leica). Special software (tissue-includer) was written in the high level language, QUIPS, which is capable of distinguishing positive and negative cells and measuring the cell size (µm^2^) and intensity (256 grey scales) of each cell in the analysis.

The acquired image was divided into 512 x 512 pixels and each pixel was expressed in square micrometers (area) after calibration with the magnification being used. The threshold for red, green, and blue (RGB) values were initially defined, each at 1–256 levels, allowing separation of 16.7 x 10^6^ color combinations. To measure CAP-18 immunoreactivity out of the total cell population, two special binary planes were applied. Binary plane 1 and 2 were detected on the processed image and gave the threshold values of the positive and total cell population, respectively. The two binary planes were displayed by color coded contour lines, positive cells were displayed with green lines and total cells with red (Fig. S1 A-D).

Immunohistochemistry slides were coded and each staining was assessed independently by two individuals in a blinded fashion, generally resulting in <10% intra-assay variation. The epithelial and non-epithelial regions were separately assessed for quantification of CAP-18 staining in each tissue section using the tissue includer function of the soft ware. The size of surface epithelium of distal colon and upper rectum was 2.02 x 10^5^ - 1.2 x 10^6^ µm^2^ and the numbers of fields taken to analyze these areas were 40 - 50 at X 400 magnification. For the Lamina propria of distal colon and rectum, 32-45 fields were taken to analyze the area of 8.67 x 10^4^ - 1.25 x 10^5^ µm^2^. The size of lung and tracheal epithelium was 7.32-8.53 x10^4^ and the numbers of fields analyzed were 28-32. The non-epithelial regions of lung and trachea were 4.39- 8.25 x 10^5^ µm^2^ which needed 90-109 fields to analyze. The average of all fields for each tissue regions was used for quantification of positive immunoreactivity relative to the total cell area of the tissue section. The results were expressed as the total positively stained area measured x total mean intensity (1-256 levels/per pixel) of the positive area divided by total cell area measured and the unit is termed as ACIA (acquired computerized image analysis) scores (Fig. S1 E).

| **A** | **B**  **D** |
| --- | --- |
| **C** |  |

**E**

| Cell area measured (μm^2^) | 11935.32 |
| --- | --- |
| Stained area measured | 1188.86 |
| % Stained area in the cell area | 9.96 |
| Mean intensity of the positive area | 89.35 |
| ACIA score | 8.9 |
| Field number | 1 |

**Figure S1.** **Measurement of immunostaining by computerized image analysis technique.** (A) Selected field of a lung biopsy section, (B) Selection of CAP-18 immunoreactive area in epithelium, (C) Selection of total cell area in epithelium, (D) CAP-18 immunoreactivity relative to total cell area in epithelium, (E) Data obtained by automated image analysis of selected field of lung tissue section.
