# Supplementary material for: Phenylbutyrate Counteracts Shigella Mediated Downregulation of Cathelicidin in Rabbit Lung and Intestinal Epithelia: A Potential Therapeutic Strategy
Source: PLoS One. 2011 Jun 3;6(6):e20637. doi: 10.1371/journal.pone.0020637 (PMC3108617; doi:10.1371/journal.pone.0020637)
Supplement: Supporting Information S2 — Analysis of butyrate in serum. (DOC) [file pone.0020637.s002.doc]

**Supporting Information S2**

for

**Phenylbutyrate Counteracts *Shigella* Mediated Downregulation of Cathelicidin in Rabbit Lung and Intestinal Epithelia: A Potential Therapeutic Strategy**

Protim Sarker^1,2^, Sultan Ahmed^1^, Snigdha Tiash^1^, Rokeya Sultana Rekha^1,2^, Roger Stromberg^3^, Jan Andersson^4^, Peter Bergman^4,5^, Gudmundur H. Gudmundsson^6^, Birgitta Agerberth^2,a^, Rubhana Raqib^1,a^

^1^International Centre for Diarrheal Disease Research, Bangladesh, 1212 Dhaka, Bangladesh; ^2^Department of Medical Biochemistry and Biophysics, Karolinska Institutet, 17177 Stockholm, Sweden  ^3^Departments of Biosciences and Nutrition, ^4^ Department of Medicine, Center for Infectious Medicine (CIM), ^5^Department of Laboratory Medicine, Division of Clinical Microbiology, Karolinska University Hospital Huddinge, Karolinska Institutet, 14186 Stockholm, Sweden; ^6^Institute of Biology, University of Iceland, 101 Reykjavik, Iceland.

**Analysis of butyrate in serum**

Blood was collected in glass tubes (kept on ice) before and 15, 30, 45 and 60 minutes after oral treatment with sodium butyrate (NaB). Serum was separated, acetonitrile was added in a 1:4 ratio and the serum aliquots were stored in glass vials at -20 °C. The butyrate analysis is based on the method by Su et. al. (Su J, Zhang N and Ho PC. Determination of tributyrin and its metabolite butyrate in Wistar rat plasma samples by gas chromatography/mass spectrometry. Rapid Commun Mass Spectrom 2004;18:2217-22) with minor modifications. The rabbit serum samples precipitated by acetonitrile were vortexed, thawed and 250 μL was taken out into a glass microvial, which was placed in an eppendorf tube and then centrifuged at 13400 rpm for 3 min. Supernatant (150 μL) was transferred to another microvial to which 50 μL acetonitrile and 4 μL 2-ethylbutyrate solution (200 μM in acetonitrile) was added. The solution was then concentrated whereupon acetonitrile was added to a final volume of 60 μL. For the standard curve and blanks, 75 μL of serum was thawed and immediately dispensed into a glass vial containing a solution of 415 μL acetonitrile to which the internal reference, 10 μL of 2-ethylbutyrate (200 μM in acetonitrile) and different known amounts from a sodium butyrate solution (50 or 200 μM in acetonitrile-water 99:1 and 96:4, respectively) had been preadded. The mixture was vortexed for 20 s and chilled for 5 min. The upper half of the mixture (250 μL) was transferred into a glass microvial, which was placed in an eppendorf tube and then centrifuged at 13400 rpm for 3 min. To another microvial (placed in an outer chromatographic vial) 200  μL of supernatant was transferred. The solution was then concentrated whereupon acetonitrile was added to a final volume of 60 μL. The samples were then analyzed by GC-MS (an Agilent HP 6890N gas chromatograph connected with an Agilent HP 5973 MSD quadropole mass spectrometer). The instrument was equipped with a 25 m, 0.20 mm i.d., 0.33 μm phase thickness HP-ultra1 column. The oven temperature program was as follows: 60 °C for 4 min, 20°/min-120° for 3 min, 80°/min-200° for 7 min, 80°/min-250 °C for 2 min. Injector temperature was 220 °C and Detector temperature was 250 °C. Helium flow was 2 mL/min and the Inlet mode used was pulsed splitless. Two samples from a treated rabbit was analyzed twice each and the concentration was determined by the linear equation obtained from a standard curve (two separate standard curves were determined, one for each analysis) done with rabbit serum samples from healthy rabbits to which known amounts of sodium butyrate (1-50 μM) was added. Four samples with serum from untreated healthy rabbits were used as blanks and analyses of these were performed after the standard curve and before the samples in order to clear the system from any butyrate contaminant arising from the spiked standards.
